# Combined Black Rice Germ, Bran Supplement and Exercise Intervention Modulate Aging Biomarkers and Improve Physical Performance and Lower-Body Muscle Strength Parameters in Aging Population

**DOI:** 10.3390/ijerph17082931

**Published:** 2020-04-23

**Authors:** Mathuramat Seesen, Warathit Semmarath, Supachai Yodkeeree, Ratana Sapbamrer, Pisittawoot Ayood, Rungnapa Malasao, Krongporn Ongprasert, Jiraporn Chittrakul, Penprapa Siviroj, Pornngarm Limtrakul (Dejkriengkraikul)

**Affiliations:** 1 Department of Community Medicine, Faculty of Medicine, Chiang Mai University, Chiang Mai 50200, Thailand; smathuramat@gmail.com (M.S.); lekratana56@gmail.com (R.S.); p_ayood@yahoo.com (P.A.); malasaor@gmail.com (R.M.); pukrongpon@gmail.com (K.O.); jerasooutch@gmail.com (J.C.); 2 Department of Biochemistry, Faculty of Medicine, Chiang Mai University, Chiang Mai 50200, Thailand; warathit_semmarath@cmu.ac.th (W.S.); yodkeelee@hotmail.com (S.Y.); 3 Center for Research and Development of Natural Products for Health, Chiang Mai University, Chiang Mai 50200, Thailand

**Keywords:** aging, intervention, black rice germ and bran, exercise, physical performance, muscle strength, inflamm-aging, biomarkers, older adults, healthy aging

## Abstract

Aging is a time-dependent functional decline in muscle mass and strength, which is reflected in poor physical performances, hormonal imbalance, and development of chronic low-grade inflammation. This study aimed to assess the effectiveness of black rice germ, bran supplement, and exercise program either alone or in combination for 24 weeks on the aging biomarkers (C-reactive protein, Interleukin-6, Insulin-like growth factor-1, and CD4:CD8 T cell ratio) physical performance, muscle strength parameters (walking speed, sit-to-stand time, grip strength) among Thai aging population. A total of 120 healthy volunteers aged 65–74 years were assigned to the exercise group (EX), black rice germ, and bran supplement (BR) group or the combination of BR and EX group (BR + EX). Over the course of the 24-week intervention, compared with baseline data (T0), the combined BR + EX intervention significantly decreased the inflammatory biomarkers (C-reactive protein and interleukin-6 levels, both *p* < 0.05 vs. T0) and significantly increased the insulin-like growth factor-1 levels (*p* < 0.001 vs. T0). Significant improvement in physical performance and muscle strength were also observed in the combined BR + EX group (decrease in sit-to-stand time and gait speed over the 24-week intervention, both *p* < 0.05 vs. T0, and trend toward grip strength improvement at *p* = 0.088 vs. T0). Overall, our results indicated a synergistic effect towards the combined intervention with the sustainable improvement in physical performances, lower-body muscle strength, and the modulation of both inflammatory and endocrine biomarkers. This study could encourage older adults to change their lifestyles to improve healthy aging and longevity.

## 1. Introduction

An increase in the human lifespan, along with a decline in fertility rates, has led to a rise in the elderly population worldwide. By 2050, the number of older persons was predicted to reach 2.1 billion or 22% of the world’s total population, which will be higher than double the size of the world’s population in 2017. Moreover, the number of elderly people is expected to be increased than people aged between 15 and 24 years in 2050 [1]. Concordantly, Thailand, which has become an aged society since 2005, is reported to have the 2nd highest proportion of the older adults in Southeast Asia. In 2018, the percentage of Thai senior citizens was 16.06% and that percentage is predicted to reach 20% by 2021 [2].

Human physiological function declines with an increase in age. Longitudinal studies have shown that with aging, most individuals tend to develop a chronic low-grade pro-inflammatory state called “inflamm-aging” and that such a state is a strong risk factor for multimorbidity, physical and cognitive disability, frailty and even death [3,4]. Aging also correlates with skeletal muscle mass decline, which usually begins to occur in the 5th decade of one’s lifespan [5]. Deficits in muscle strength, functional and balance impairment, disabilities, and higher risks of falling are all consequences of muscle loss in the elderly. Notably, the decrease in muscle strength in older adults not only relates to physical disability but also increase the incidences of mortality [6,7,8,9].

Altered intercellular communication (innate immune systems hyperactivation leading to an increase in circulatory levels of proinflammatory cytokines and changes in neurohormonal signaling such as GHRH-GH-IGF-1 axis, etc.) and the decline in function of adaptive immunity (cellular senescence) are essential hallmarks of aging that are believed to be triggered by a lifelong accumulation of the oxidative damage that occurs during the aging process [10,11,12,13]. Oxidative stress elicits an amplification of the release of cytokines that has been reported to be correlated with losses in muscle mass and strength with regard to the Reactive Oxygen Species (ROS) production leading to further declines in muscle performance and muscle mass. Furthermore, elevated oxidative stress is also associated with a decline in physical performance, such as walking speed in the elderly [14,15,16]. With regard to inflammatory cytokines, our previous study reported that the elevated levels of Interleukin-6 (IL-6) and C-reactive protein (CRP) were associated with a hazardous ratio for decreased grip muscle strength in older adults [17]. Moreover, the high levels of IL-6 over time is considered a potential condition of impending and evolving multimorbidity [18]. Additionally, changes in the endocrine system that typically occur during the aging process, such as an imbalance in Insulin-like Growth Factor-1 (IGF-1) hormonal levels, would also likely to be an important factor in an accelerated decline in muscle mass and strength in the elderly [19,20]. Therefore, based on the previous findings, an intervention that could target low-grade chronic inflammation and also restore hormonal dysregulation in aging individuals could result in improving physical performances and muscle strength as well as to reduce the relevant risk factors for chronic diseases. Notably, the latter is considered a significant pathological process of multimorbidity and unhealthy aging.

Exercise was previously reported to decrease various biomarkers of inflammation in older adults (CRP and cytokines such as IL-1β, IL-6 etc.) and to modulate the hallmarks of aging such as, by activating the nutrient-sensing pathway via an increase in GH and/or IGF-1 levels [21,22]. Moreover, exercise intervention has been proposed as an effective method to enhance muscle strength, physical activity, and to prevent falling in the elderly [23,24,25]. Besides, many nutritional supplements were indicated to amplify the effects of exercise on physical performance and muscle strength in the elderly. However, the previous studies were only focused on positive impacts on the metabolism of amino acids, proteins, and vitamin D [26,27]. In recent years, the aims of clinical intervention among aging individuals have not only targeted the physical performances and muscle strength of aging subjects, but they have also been designed to target the inflamm-aging phenomenon as a preventative measure of chronic diseases. Zinc (Zn), Resveratrol, Epimedium total flavonoids, and Icariin are the clinical drugs that have been commonly used for this purpose as they are also considered to be effective, safe, and non-toxic to the elderly [28,29,30,31,32,33]. Recently, pigmented rice varieties, such as red rice, purple rice, and black rice, have received increased attention for their various phytochemical compounds including phenolics, flavonoids, tocols, and sterol derivatives. These bioactive compounds are primarily located in the outer layer of the rice grain, germ, and bran [34,35,36,37]. Black rice germ and brans are characterized by the presence of anthocyanin phytochemicals such as cyanidin-3-O-glucoside (C3G) and peonidin-3-O-glucoside. These phytochemicals have been reported for their antioxidant capacity and anti-inflammatory activity [38,39,40]. Anthocyanin-rich black rice extract was found to possess anti-inflammatory effects conducted on a macrophage cell line and reduced the inflammatory response induced by lipopolysaccharide (LPS) treatments. It has also been found to mechanistically diminish the secretion of pro-inflammatory cytokines (TNF-α and IL-6) via the inhibition of the mitogen-activated protein kinase (MAPK) signaling pathway. This can lead to a decrease in nuclear translocation of NF-κB and Activator Protein 1(AP-1) [41,42]. Additionally, the anti-inflammatory effects of black rice extract were believed to be related to other compounds present in the extracts besides anthocyanins, potentially acting in an additive or synergistic way. Moreover, the anti-inflammatory capacity of black rice is potent regardless of the thermal or cooking process [43,44,45].

To the best of our knowledge, there has been no study of the effectiveness of the supplements, that aiming to decrease systemic inflammation and restore the anabolic hormone levels in combination with an exercise program for the improvement of physical performance, muscle strength, and blood-based aging biomarkers in the elderly. Therefore, this study was conducted to evaluate the effectiveness of black rice germ and bran supplement, exercise program, and a combined intervention on various aging biomarkers, physical performance and muscle strength in community-dwelling elderly. This was done in order to achieve the goal of establishing a healthy aging policy in many developed and developing countries including Thailand.

## 2. Materials and Methods

### 2.1. Study Design and Participants

**Figure 1:**
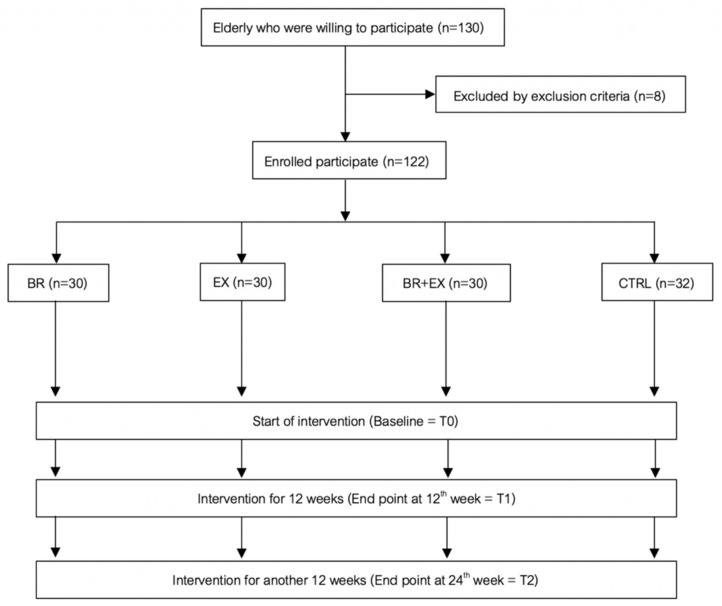
Diagram of sample selection and intervention study design for the aging population (BR = Black rice germ and bran supplement intervention group, EX = Exercise intervention group, BR + EX = Combined intervention of supplement intake and exercise intervention group and CTRL = Control group).

This prospective study was conducted from September 2018 to May 2019, starting from the recruitment of participants up until the last time-point of the intervention follow-up phase. Participants were community-dwelling older adults aged 65–74 years who lived in Pasang District in Lamphun Province and in Khua Mung District in Chiang Mai Province. Both provinces are located in the northern part of Thailand. Per the aging population of each region of Thailand, the north region is recognized as having the highest proportion of aging citizens in the country. Moreover, with regard to the statistics of the specific provinces included in this study, Chiang Mai Province is currently recognized as having the third largest elderly population. At the same time, Lamphun Provinces is ranked as having the highest aging index in northern Thailand [2,46]. The elderly who lived in the study area during the course of the study period, and who were able to communicate in Thai, were asked to participate in this study. These stipulations were considered as one of the inclusion criteria. 130 older adults were randomly recruited from 526 participants in the enrolling list. 8 participants were excluded from this study based on the exclusion criteria, which included disabilities of limbs; walking abnormalities; having undergone surgery in the 6 months prior to the study; diagnosis of a neurological abnormality; diagnosis of severe depression; blindness or severe vision impairment; and diagnosis of cancer. The elderly who participated in the community exercise groups were also excluded. Finally, 122 subjects participated in the study in which they were further divided into four groups and assigned to different intervention programs (Figure 1).

At baseline, participants underwent a detailed health assessment that served as a screening method. The screening process was overseen by well-trained researchers and doctors working at the Department of Community Medicine, Faculty of Medicine, Chiang Mai University. The screening method was comprised of questionnaires, physical measurements, and a review of the medical records that were held by the general practitioners working in the area. Participants were asked whether a doctor had ever told them that they had any of the following conditions: myocardial infarction, diabetes or high blood sugar, high blood cholesterol, osteoarthritis, or osteoporosis.

### 2.2. Intervention Programs

Participants were divided into four groups using a matched-pair design. These groups were comprised of 3 intervention groups and a single control group. Sample selection was carefully conducted with regard to the generalization of the population of this study group. The black rice germ and bran intake group (BR) was assigned to drink black rice germ and bran instant powder (a commercially available product with Thai FDA approval). The nutritional values of black rice germ and bran powder supplement are provided in Table 1. The dosage consisted of 10 g of black rice instant powder per day providing a total of 300 mg anthocyanins/day. This was found to be sufficient to promote immunomodulatory effects [47]. The amount of total anthocyanin content in black rice instant powder, which was provided to subjects daily in this study, corresponded to 32 mg/g of black rice powder, and this amount was considered an appropriate dose for the health-promoting purposes of this study [40,42]. Participants were instructed to mix a package of black rice instant powder with 150 mL. of hot water and to drink the prepared beverage after breakfast and after dinner. If subjects had forgotten to consume the beverage after the meal, participants could drink the prescribed beverage just as soon as they remembered to drink it. The compliance of participants in this study was measured by the follow-up of residual package of black rice germ and bran powder count and return of the empty package. Briefly, participants were asked to return the empty packages of the consumed black rice germ and bran powder of the past 2-week along with receiving the new set of black rice powder package for the next 2-week.

The exercise group (EX) followed a regimen that was adapted from an exercise training program for older adults as had been previously described [48]. Subjects in this group were assigned to do certain physical activities following a prescribed physical therapist demonstration three times a week consecutively for 12 weeks. The exercise activities took place at Pasang and Khua Mung District local Health-Promoting Hospitals. The exercise intervention consisted of 3 sessions involving a warm-up period for 5 min, exercise training for 40 min, and a cool down period for a subsequent 5 min. The warm-up session included stretching of the neck, arms, wrists, back, tight, knees, and then involved breathing exercises. The exercise training session included nine strengthening movements (Sit-to-stand, Knee raise standing, Squats to chair, Step back lunges, Step up standing, Step forward standing, Step backward standing, Step to side standing, and 3 steps standing) and eight balance exercise movements (Hell-to-toe standing, Side leg raise, Heel raise, Calf and toe raise, One leg standing, Double legs stance with eyes closed, Double legs stance on unstable surfaces, and Crossover). Each movement was performed ten times for two rounds with a 2-min break per round. For the walking and standing balance exercise, participants were assigned to walk 20 steps and then to stand for 15 s in each round. For the cool-down session, the movements were similar to those of the warm-up sessions. For another 12 weeks, the exercise intervention was carried on as a home-based exercise regimen. Participants were instructed and assigned to continue a similar exercise program at their own home. The levels of compliance and willingness of all participants were measured by the record of participation, which refer to as the presence of participants at the place that exercise program being held and at their home during home-based exercise period. The data of participation were record by local health-promoting hospital staff and calculated as the percentage of participation. During the intervention, participants were encouraged and followed-up upon by doctors from the Department of Community Medicine and volunteer officers from the local health-promoting hospitals.

Participants in the combined black rice germ and bran intake and the exercise intervention (BR + EX) groups were assigned to drink black rice instant powder at an amount of 10 g per day for 24 weeks and participated in the exercise intervention at the local health-promoting hospital for 12 weeks and continued with the home-based exercise regimen for another 12 weeks. The control group was given health education instruction in terms of nutrition and general physical activities for the elderly without an intervention program.

### 2.3. Outcome Measurements

All outcome measurements were conducted at the baseline (T0), at the end of the 12th (T1) and 24th (T2) weeks of the intervention program for all participants of each intervention groups. Physical Performance was assessed by gait speed on a 6m course. Participants were instructed to walk at their normal pace. The timing was stopped when each participants’ foot touched the floor at the end of the marked path [49]. Handgrip strength was measured as an estimate of upper body muscle strength using a digital hand dynamometer (TAKEI T.K.K.5401^®^, Takei Scientific Instruments Co.,Ltd, Tokyo, Japan). Grip strength is considered a valid and reliable method to measure muscle strength [50,51]. Each participants’ dominant hand was tested for grip strength. Participants sat upright with their shoulders slightly adduct, elbows at 90 degrees of flexion, and wrists kept in a neutral position. Participants were asked to maintain this position during the test and to squeeze the dynamometer using their maximal strength for 2–3 s. The procedure was performed three times, with a rest period of at least 15 s between each measurement. The mean value of 3 trials was reported. The Sit-to-Stand test was based on the Five-Times-Sit-to-Stand test (FTSS) that used as a measurement of lower extremity functioning [52]. Participants were requested the FTSS on a 45-cm-tall chair with the arms folded across the chest. The test involved standing up from a sitting position and sitting down five times without pushing off. Timing began when the rater spoke the word “go” and stopped when the participant’s buttocks reached the seat following the fifth stand. The raters required participants to stand and sit five times as quickly as possible without physical assistance. Frailty measurement was also defined for this population for the purposes of internal control using the Fried’s model of five phenotypic frailty criteria (weight loss, exhaustion, low physical activity, weakness, and slowness) [53]. Participants were classified as non-frail (those who met none of the phenotypic criteria), pre-frail (those who met one or two of the phenotypic criteria), and frail (those who met three or more data points of the phenotypic criteria). Depression was assessed by a nine-question assessment tool for depression severity (9Q) with scores ranging from 0 to 27. A score of 7 or over indicated depressive symptoms. Cognitive status was assessed by the Mini-Mental State Examination-Thai version (MMSE-Thai) with scores ranging from 0 to 29 [54]. A score of 17 or lower in older adults, who had received primary education, indicated cognitive impairment.

Blood collection was performed after an overnight fast, wherein 15 mL blood was drawn from the antecubital vein between 7:00 and 9:30 am. Great attention was being paid to getting blood samples to the laboratory as fast as possible, and samples were received for processing within 3 h after the blood collection procedure. EDTA plasma and serum samples were obtained and analyzed for complete blood count (CBC), lipid profile (total cholesterol, triglycerides, HDL-cholesterol and LDL-cholesterol), liver enzymes (AST & ALT), kidney function tests (BUN, Creatinine). Plasma contained in the NaF tube that was analyzed for fasting blood glucose. Aliquots of serum were kept at −80 ℃ in frozen-stored cryogenic tubes for further used in aging biomarkers measurements, including IL-6, CRP, and IGF-1 levels. The serum levels of these biomarkers were measured with ELISA kits (IL-6; Biolegend, USA and CRP & IGF-1; Abcam, UK). For immunosenescence biomarkers, the lymphocyte immunophenotyping and blood samples were analyzed by three-color direct immunofluorescence (Tri-test) flow-cytometry (Becton Dickinson FACScan Flow Cytometer) for CD4 fluorescein isothiocyanate (FITC)/CD8 phycoerythrin (PE)/CD3 peridinin chlorophyll protein (PerCP). A fluorescence-labeled antibody was obtained from BD Biosciences (Oxford, UK).

### 2.4. Sample Size Calculation and Statistical Analysis

The sample size was calculated based on the sit-to-stand test with a confidence interval of 95%, and a bilateral hypothesis test with a significance level of 0.01 and a power of 90%. A total of 26 subjects were needed for each group. A noncompliance rate of 20% for the intervention group and a drop-out rate of 20% were considered for a final sample size of 120 participants was decided (at least 30 individuals per group).

Descriptive analysis was performed on the demographic and general health data, as well as frailty status, depression score, and cognitive function. For continuous variables, the Kolmogorov-Smirnov test was used to assess the normality of distribution, and the data were expressed as the mean ± SD or ± standard error of the mean (SE). Numbers, percentages, and median were used for categorical variables. The within-group comparisons (among T0, T1, and T2 weeks) were made using repeated measured ANOVA and Bonferroni tests for continuous data and the Friedman test for categorical data. For each parameter, prior to the comparison, the means values of baseline data (T0) obtained from 4 different intervention groups were assumed as non-significant difference by using one-way ANOVA and Kruskal–Wallis H test for continuous data and categorical data, respectively. The significant level was set at *p* < 0.05 or < 0.01. All analyses were performed using SPSS 21.0 (IBM, New York, USA). Any missing data were not replaced.

### 2.5. Ethical Consideration

All subjects gave their informed consent for inclusion before they participated in the study. The study was conducted in accordance with the Declaration of Helsinki, and the protocol was approved by the Ethics Committee of Faculty of Medicine, Chiang Mai University (Ethical number: COM-2561-05171: Date of approval; August 22nd, 2018).

## 3. Results

### 3.1. Baseline Descriptive Data of Sociodemographic Characteristics and Health Profile of the Aging Population

The baseline characteristics of 122 participants are shown in Table 2. No significant differences in the baseline characteristics were observed between the intervention groups with respect to the sociodemographic data or health profiles (*p* > 0.05). At the baseline, screening for frailty phenotypes using Fried’s model found no significant differences in both the mean frailty score and percentage of frail subjects among the intervention groups (*p* > 0.05). The Blood-based health profile including CBC, total cholesterol, liver enzymes (AST & ALT), kidney function tests (BUN, Creatinine), fasting blood sugar as well as aging biomarkers (IL-6, CRP, IGF-1), and immunosenescence biomarkers also found no significant differences among the intervention groups at the baseline point (T0) (data are shown as the baseline value or T0 column of 24-week intervention in Table 3 and Table 4, all *p*-values > 0.05).

### 3.2. Changes in Blood-Based Health Profile and Aging Biomarkers during 24-Week Intervention Period

#### 3.2.1. Changes in Blood-Based Health Profile

For the duration of 24 weeks from the start of the intervention program, a total of 120 subjects (men, *n* = 45.; women, *n* = 75) had completed the intervention program, while 2 participants withdrew from the study before the intervention had finished due to the personal health reasons or a lack of willingness to continue participating in the program.

Prior to determining the improvement of the aging biomarkers and the immunosenescence biomarkers, we first determined the effect of 24-week intervention programs on the general blood health profiles of the participants of 4 different groups by measuring the complete blood count-, lipid profile-, fasting blood glucose-, liver enzyme- and kidney function-parameters as is shown in Table 3 and Table 4. Over the course of the 24-week period, each intervention group, including the CTRL group, reported no significant effects in terms of CBC (RBC count, hemoglobin, platelet count, WBC count, and differential count, data not shown), kidney function, and liver enzyme parameters when compared with the T0 value in each of their respective groups (T1 vs. T0 and T2 vs. T0, all *p* > 0.05). However, a significant reduction in blood total cholesterol and triglycerides of the participants in the black rice alone (BR) group was observed in the 24th week (T2 vs. T0, *p* < 0.05). In addition, a significant decrease in the fasting blood sugar level was observed in participants of the combined intervention (BR + EX) group at both the 12th and 24th weeks when compared with the baseline data (T1 vs. T0 and T2 vs. T0, both *p* < 0.01). Overall, all of the intervention programs had no adverse effects with regard to the general health profile of the participants in each group over the period of a 24-week course.

#### 3.2.2. Changes in Aging Biomarkers

**Figure 2:**
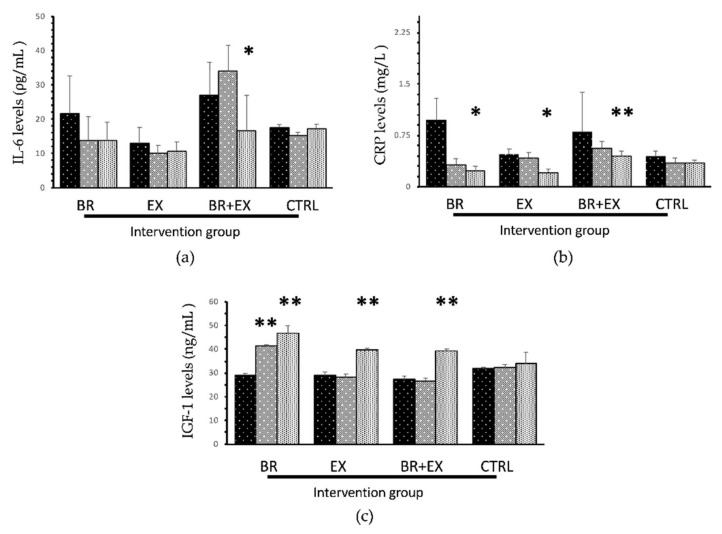
Changes in aging biomarkers in all 4 different intervention groups during the 24-week intervention period: (**a**) Interleukin-6 levels; (**b**) C-reactive protein levels; (**c**) Insulin-like growth factor-1 levels (data were expressed as mean ± SE, BR = Black rice germ and bran alone group, EX = Exercise alone group, BR + EX = Combined group and CTRL = Control group) * *p* < 0.05, ** *p* < 0.001 vs. baseline with statistical significance using repeated measured ANOVA.

**Figure 3:**
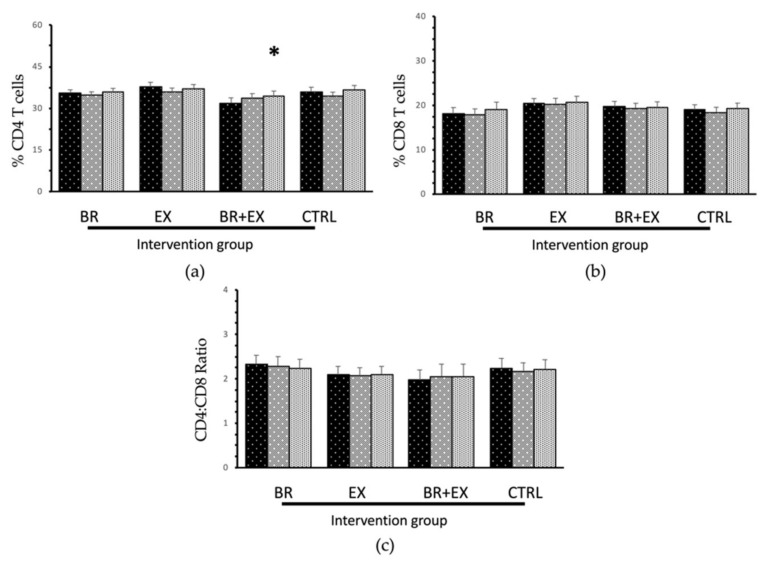
Changes in immunosenescence biomarkers in all 4 different intervention groups during the 24-week intervention period: (**a**) %CD4 + T cells; (**b**) %CD8 + T cells; (**c**) CD4^+^/CD8^+^ T cell ratio. (data were expressed as mean ± SE, BR = Black rice germ and bran alone group, EX= Exercise alone group, BR + EX = Combined group and CTRL = Control group). * *p* < 0.05 vs. baseline with statistical significance using repeated measured ANOVA.

To determine the effects of the 24-week intervention programs on changes in aging biomarkers in each group, we detected the serum levels of the inflammatory biomarkers (IL-6 and CRP), endocrine biomarker (IGF-1) and immunosenescence biomarker (CD4^+^/CD8^+^ T cell ratio). The aging biomarkers changed within each group (BR, EX, BR + EX, and CTRL group), are shown in Figure 2 and Figure 3 and re-tabulated in Table 5. Compared with the baseline data(T0), the black rice supplement alone (BR) group showed a significant decrease in serum CRP levels at the 24th week (T2 vs. T0, *p* < 0.05) together with a significant increase in serum IGF-1 levels at both the 12th and 24th week (T1 vs. T0 and T2 vs. T0, respectively with *p* < 0.001). Serum IL-6 levels, another inflammatory biomarker, was found to have decreased in a borderline significance manner over the period of the intervention (*p* = 0.058) in BR alone group. Exercise intervention alone (EX) group also followed the same trend of significant changes as did the BR group, with CRP levels showing a decrease, while the IGF-1 level increased when compared with their respective baseline of each parameter. However, significant differences were not observed until the 24th week of the intervention program (T2 vs. T0, both *p* < 0.001).

As expected, the combined intervention (BR + EX) group showed remarkable changes at the 24th week of the intervention program. When compared with the baseline data, significant decreases in serum levels of both inflammatory biomarkers were observed in the combined intervention group (IL-6 and CRP, both *p* < 0.05) together with a significant increase in both serum IGF-1 levels and the % CD4 T cells (*p* < 0.001 and *p* < 0.05, respectively). Surprisingly, all of the intervention groups (BR, EX, and BR + EX groups) revealed no significant changes in the CD4:CD8 T cell ratio biomarker (all *p* > 0.05 vs. baseline). Additionally, there was no significant change being observed among all parameters of the control (CTRL) group during the 24-week of intervention program (all *p* > 0.05). Overall, BR and EX alone groups displayed significant effects in terms of decreases in CRP levels and increases in IGF-1 levels at the 24th week when compared with the T0 week of the intervention. While the combined BR + EX intervention had significant effects in terms of decreases in both inflammatory biomarkers’ levels (IL-6 and CRP) and increases in the endocrine biomarker’s levels (IGF-1) at the 24th week of the intervention. Moreover, when comparing the changes in aging biomarkers between the combined BR + EX group and BL alone group, the combined group revealed significant effects on the aging biomarkers (IL-6, CRP, and IGF-1) that differed from the BL group (mean differences of T1-T0 and T2-T0, all *p* < 0.05; data not shown).

### 3.3. Changes in Physical Performance and Muscle Strength during 24-Week Intervention Period

**Figure 4:**
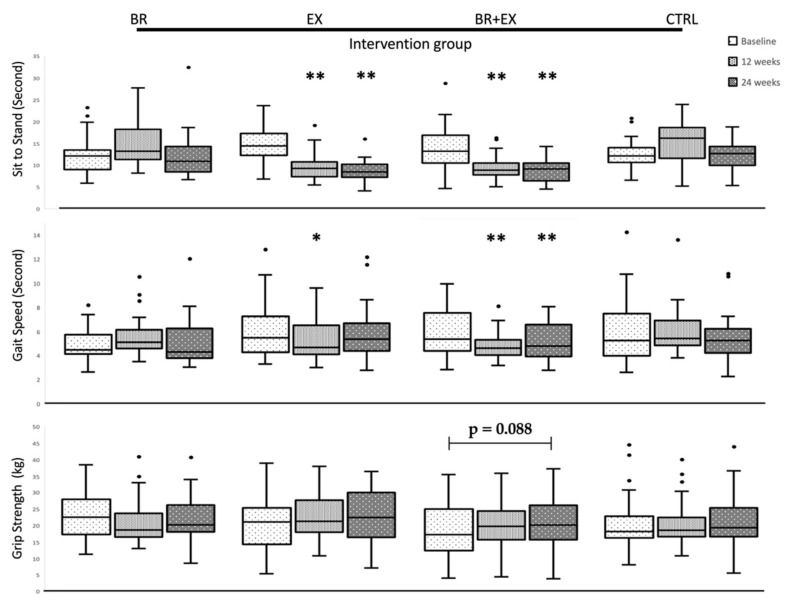
Changes in Sit to Stand (Upper), Gait Speed (Middle) and Grip Strength (lower) in all 4 different intervention groups during the 24-week intervention period (BR = Black rice germ and bran alone group, EX = Exercise alone group, BR + EX = Combined group, and CTRL = Control group) * *p* < 0.05, ** *p* < 0.001 vs. baseline with statistical significance using Friedman test.

The physical performance and muscle strength changes within each group (BR, EX, BR + EX, and control group) are shown in Figure 4, and the data were re-tabulated and presented in Table 6. Compared to the baseline data (T0), the exercise alone (EX) group showed the significant improvement in sit-to-stand time at both the 12th (T1) and 24th (T2) weeks of the intervention period (*p* < 0.01), as well as for the gait speed at the 12th week of the intervention period (*p* = 0.019). Moreover, the combined intervention (BR + EX) group reported significant improvement of both sit-to-stand time and the gait speed at the 12th and 24th weeks of the intervention program when compared with the baseline (T1 vs. T0 and T2 vs. T0, both *p* < 0.01). There was no significant change in grip strength being observed in all of the intervention groups. Nevertheless, Participants in the BR + EX group showed a trend towards the improvement in grip strength with borderline significance (*p* = 0.088). For the supplement intervention group, the black rice alone (BR) group reported no significant effects on either physical performance or muscle strength parameters (all *p* > 0.05), except for the combined with exercise group (BR + EX) as has been previously described. No significant changes were observed among all parameters in the CTRL group during the 24-week program of intervention (all *p* > 0.05). Moreover, when comparing the significant changes in physical performances and muscle strength parameters between the combined BR + EX group and the EX alone group, the combined intervention had a significant mean difference on both STS time and gait speed time when compare with EX alone group at the 12th and 24th weeks of the intervention period (T1-T0 and T2-T0, *p* < 0.01 and *p* < 0.001, respectively; data not shown). Overall, the BR + EX and EX alone groups had shown significant improvements in terms of physical performance and lower-body muscle strength (as evidenced by the reduction of both STS time and gait speed time). Notably, the combined group showed significant improvement effects in terms of physical parameters that differed from the EX alone group.

## 4. Discussion

The aging process that turn into a negative effect is defined as an aging individual who would have an increased risk of acquiring chronic diseases including cardiovascular diseases, type 2 diabetes, cancer, and neurodegenerative conditions [55,56]. One of the possible etiologic factors that can lead to unhealthy aging is believed to be related to the consequences of an accumulation of damage that trigger chronic inflammation, which is referred to as inflamm-aging [4,57]. The inflamm-aging determinants involve an increase in inflammatory cytokines levels such as IL-6 and CRP. Another factor is an imbalance in hormonal levels in the somatotropic-axis, such as growth hormone and IGF-1, which are considered to be some of the biomarkers for the disturbance of the endocrine systems during aging [58,59]. All of these phenomena affect the physiologic changes and contribute to the symptoms of vulnerability in aging individuals [60,61].

Musculoskeletal and endocrine systems are responsible for the changes in physical performances and muscle strength during the aging process. The weakness and the slowed motor performance are considered as the cardinal features of sarcopenia, which refers to a loss in muscle mass and strength [62]. The pathological effects of sarcopenia included changes in α-motor neurons, type I muscle fibers, muscular atrophy, poor nutrition, and frailty syndrome [5,59,63]. Growth hormone and IGF-1 are hormones that are essential to skeletal muscle metabolic regulation. Lower levels of IGF-1 are associated with lower strength, decreased mobility, and increasing levels of the markers of inflammation, including CRP and IL-6 levels in older adults [19,64]. A combination of low IGF-1 and high IL-6 levels in a cohort of community-dwelling older adults conferred a high risk for progressive disabilities and death that was greater than the effect of either of these two factors alone, suggesting an additive effect [18,65]. Similarly, Roubenoff et al., showed that increased cellular production of IL-6 and decreased cellular production of IGF-1 were associated with increased death rates over a four-year period in a cohort of community-dwelling older adults [66].

This study was the first report to display the effects of combining both exercise program and phytochemical supplement interventions that target the inflamm-aging phenomenon and improve the physical health in the aging population. Our results showed that the 24-week exercise program (EX) intervention program alone had an effect on the increasing levels of IGF-1 in the serum of aging individuals. Moreover, this intervention had a significant impact on improving the sit-to-stand- and gait speed-time parameters, which can reflect the overall physical performance and lower-body muscle strength. Nevertheless, improvements in gait speed time could not be sustained until the end of the 24th week (T2). Regarding the exercise intervention, our results are consistent with those of a previous study, which indicated that a multicomponent exercise intervention regimen had a beneficial effect on muscle strength and muscle power as measured by gait speed and sit-to-stand performance in the elderly subjects [67]. Additionally, exercise is an interventional modality that has most consistently shown beneficial physiologic impacts on musculoskeletal, endocrine, and immune systems as evidenced by a large number of trials that have demonstrated the positive impacts of an exercise intervention on muscle strength and functional mobility [68]. Shahar et al., had reported a significant increment of muscle strength and body composition using a 12-week exercise program developed for the sarcopenic elderly [27]. Candow et al., also reported that 22 weeks of whole-body resistance exercise training program for healthy older males, aged 60–71 years, was sufficient to overcome the age-related deficits in upper and lower body strength [69].

Additionally, the black rice germ and bran supplement (BR) intervention alone could significantly decrease CRP levels in the serum, while inversely increasing serum IGF-1 levels in the aging participants of this group at the 24th week of intervention. Currently, in addition to exercise, natural products are now used as supplements that offer a number of health benefits. These beneficial effects included correcting nutritional deficits, anti-inflammatory, and antioxidant properties, as well as other potential benefits. [33,68,70,71,72]. Black rice germ and bran, by-products of rice, contain a high amount of anthocyanin phytochemicals that possess strong antioxidant and anti-inflammatory properties [41,42]. Moreover, a previous study has also found that cranberry juice beverage containing anthocyanins could lower the CRP levels in human study [73]. Apart from inflammatory biomarkers, our results also showed a significant increase in IGF-1 levels upon consumption of the BR supplement. This outcome coincided with the findings of a previously published report that showed a significant increase in IGF-1 expression from berries’ polyphenols consumption, which can result in the improvement of motor and cognitive performances in aging rat model [74]. In addition to the aging biomarkers, BR supplement alone could significantly reduce the total cholesterol and triglyceride levels in the blood. The results are in agreement with those of previous studies with regard to the lipid-lowering effects of anthocyanin-rich products [75,76]. It is no surprise that neither physical performance nor muscle strength parameters were found to be non-significantly improved from the BR alone intervention. As anthocyanins found in the pigmented rice are usually focused on their beneficial effects in terms of endocrine modulation and anti-inflammation properties. Unlike the black rice germ and bran in our study, the macromolecules used in other studies focused on certain supplemental proteins or branched-chain amino acids for an indication of increased muscle protein synthesis and to maintain the muscle mass and muscle function in the elderly [27,77,78,79]. Moreover, there has been no report on the beneficial effects of macromolecules on inflammatory and endocrine biomarkers in aging populations. For these reasons, black rice germ and bran supplement have made their way onto our list as the potential candidates for inflamm-aging intervention.

Lastly, indeed, the combined exercise program and the black rice germ and bran supplement (BR + EX) intervention program was able to improve older adults’ lower-body muscle strength and physical performance parameters. This was done by measuring the improvements in sit-to-stand and gait speed performances. Additionally, this combined intervention also showed remarkable effects that were significantly different from the EX alone intervention. This level of improvement was sustained until the end of the experiment (at the 24th week). Our result was in agreement with those of previous studies wherein the physical performance and muscle strength in the exercise program and nutritional supplement combination showed the higher effectiveness when compared with either the exercise or the supplement alone [80,81]. Moreover, with regard to changes in the aging biomarkers, our results showed that the combined intervention was able to significantly modulate those aging biomarkers including, by enhancement of the IGF-1 biomarker and by lowering the inflamm-aging biomarkers (both CRP and IL-6) throughout the course of the 24-week intervention. With the addition of lowering the IL-6 inflammatory cytokine levels and sustainable effects in terms of physical performances, lower-body muscle strength and aging biomarkers modulation, we hypothesize that there are synergistic effects to the exercise program and the black rice germ and bran supplement. Previously, many studies have reported on the pronounced anti-inflammation properties of black rice. Black rice extract was found to be able to inhibit the cytokines production from the immunocompetent cells [82], moreover, Limtrakul et al., reported that the anthocyanin-rich fraction obtained from black rice could significantly suppressed the inflammatory response in macrophage cells via the inhibition of IL-6 and TNF-α secretions [42]. Additionally, with regard to exercise and diet lifestyle, a 15-week course of daily exercise and a caloric-controlled diet could significantly lower the levels of inflammatory biomarkers, CRP and IL-6, and increase insulin sensitivity [83]. These beneficial effects are also in agreement with the findings of our study since we also reported significant decreases in inflammatory biomarkers together with a significant reduction in fasting blood sugar in our combined intervention group. Unfortunately, over the 24-week period of all of the intervention groups, we did not find any significant improvement in the immunosenescence biomarker as measured by the CD4:CD8 ratio. However, since this study was focused on the early-aging population who were between 65–74 years old, and the CD4:CD8 ratio at the baseline (T0) was within the normal reference value [84,85]. Therefore, this parameter may not be considered a risk factor for people in this age range.

This study used the combined black rice germ and bran supplement and the 12-week exercise program as a combined ideal choice for intervention to improve physical performances, muscle strength, and aging biomarkers. The aim of the researchers in this study was placed on identifying significant physical improvements as a consequence of inflammatory- or endocrine-related systemic biological changes. These changes have been recognized as hallmarks of aging in terms of deregulated nutrient sensing (changes in somatotropic IGF-1-axis) and the alter intercellular communication (low-grade chronic inflammation from the increased threshold of proinflammatory cytokines) [10]. The hallmarks above share an interconnected pathway from the perspective of “the driver of inflamm-aging” mechanistically via the dysregulation of the NF-kB signaling pathway leading to an excessive production of ROS and an accumulation of senescent cells [33,56,60]. Additionally, the levels of anabolic hormones, such as IGF-1, that decline significantly with aging have also been found to be inversely associated with the increased inflammatory biomarkers. This outcome might lead to an increase susceptibility to chronic diseases in those aging individuals [18,19,65]. However, the potential crosstalk mechanism of the inflamm-aging and somatotropic-axis pathways needs to be further investigated.

## 5. Conclusions

Overall, our data revealed the synergistic effects of black rice germ and bran supplement when combined with exercise program through the inflammatory and endocrine biomarkers modulation together with improvements in physical performance and lower-body muscle strength. This could encourage aging individuals who would like to improve their health-related lifestyle with regard to physical activity and diet-related behavior. As a consequence, these modifications to lifestyle could lead to healthy aging and improved longevity in older adults. Unfortunately, over the course of a 24-week intervention program, there was no significant improvement in grip strength, which considered as the representative of upper-body muscle strength. We should notify that, this study lack of grip strength improvement. This could be due to the insufficient intensity of exercise training program to produce the adaptation. Perhaps, with more intensive training program probably could improve grip strength and change of some results. Moreover, the extension of either the sample size of the aging population or an extension in the exercise program, and/or the black rice supplement duration period, may provide a more accurate and significant outcome for a future study.

## Figures and Tables

**Table 1:** Nutritional value information and bioactive ingredients of the black rice germ and bran supplement.

Average Nutrition Information	100 g
Energy (Calories)	300 Kcal.
Total Fat	0 g
Cholesterol	0 g
Total Carbohydrate	80 g
Dietary Fiber	8 g
Protein	10 g
Sodium	0.015 g
Vitamin B1	2 g
Iron	1.5 g
Bioactive ingredients	g/100 g
Phenolic compounds	5.21 ± 0.13
Total flavonoids	5.14 ± 0.06
Tocopherol	0.013 ± 0.002
Anthocyanins	3.2 ± 0.07

**Table 2:** Baseline (T0) descriptive data of sociodemographic characteristics and health profiles of participants.

Characteristics	BR Group (*n* = 30)	EX Group (*n* = 30)	BR + EX Group (*n* = 30)	CTRL Group (*n* = 32)	*p*-Value
Gender, *n* (%)					0.653
Male	11 (36.7)	14 (46.7)	12 (40)	10 (31.3)	
Female	19 (63.3)	16 (53.3)	18 (60)	22 (68.8)	
Age, mean ± SD	68.80 ± 2.82	68.17 ± 2.65	68.20 ± 2.41	69.06 ± 2.74	0.464
Marital status, *n* (%)					0.759
Single	3 (10)	2 (6.7)	2 (6.7)	5 (15.6)	
Married	19 (63.3)	21 (70)	17 (56.7)	22 (68.8)	
Separate/ divorce/ widow	8 (26.7)	7 (23.3)	11 (36.7)	5 (15.6)	
Number of comorbidities (mean ± SD)	1.10 ± 0.88	0.96 ± 0.85	1.10 ± 1.14	1.0 ± 1.27	0.942
Alcohol at present, *n* (%)	3 (10)	3 (10)	4 (13.3)	5 (15.6)	0.883
Smoking at present, *n* (%)	2 (6.7)	3 (10)	4 (13.30	3 (9.4)	0.863
Depression score > 7, *n* (%)	0	3 (10)	3 (10)	3 (9.4)	0.365
Frailty N (%)	14 (46.7)	14 (46.7)	15 (50)	16 (50)	0.988
Frailty score, mean ± SD	1.43 ± 1.56	1.60 ± 1.77	1.66 ± 1.72	1.71 ± 1.80	0.239

BR = Black rice germ and bran supplement intake intervention group, EX = Exercise intervention group, BR + EX = Combined intervention of supplement intake and exercise intervention group and CTRL = Control group). Statistical significance at *p* < 0.05 using Kruskal–Wallis H test for categorical data and one-way ANOVA for continuous data.

**Table 3:** Changes in general blood-based health profile parameters (kidney function, liver enzymes, and fasting blood sugar) in all 4 different intervention groups during the 24-week intervention period.

Parameter	Mean (SD)															
	BR Group (*n* = 30)				EX Group (*n* =30)				BR + EX Group (*n* =28)				CTRL Group (*n* = 32)			
	T0	T1	T2	*p*-Value	T0	T1	T2	*p*-Value	T0	T1	T2	*p*-Value	T0	T1	T2	*p*-Value
**BUN** (mg/dL)	14.11 (5.15)	14.11 (5.32)	15.09 (5.46)	0.329	15.30 (4.86)	15.68 (4.58)	16.34 (6.27)	0.545	13.40 (4.67)	13.91 (5.56)	14.49 (4.57)	0.240	13.60 (3.06)	13.55 (3.87)	14.84 (4.56)	0.167
**Creatinine** (mg/dL)	0.92 (0.28)	0.89 (0.23)	0.87 (0.21)	0.054	0.91 (0.24)	0.90 (0.23)	0.95 (0.31)	0.052	0.89 (0.31)	0.88 (0.27)	0.89 (0.30)	0.355	0.87 (0.22)	0.86 (0.22)	0.85 (0.22)	0.108
**AST** (U/L)	23.67 (11.5)	24.40 (14.3)	24.10 (9.0)	0.526	22.97 (10.7)	22.60 (7.8)	24.60 (11.0)	0.156	22.86 (7.7)	24.18 (10.2)	23.32 (8.4)	0.681	21.32 (5.3)	21.19 (5.7)	22.23 (7.6)	0.732
**ALT** (U/L)	20.17 (14.0)	21.43 (17.0)	19.87 (10.4)	0.514	19.00 (12.8)	18.13 (10.0)	16.60 (10.6)	0.101	16.71 (7.5)	18.00 (9.6)	14.71 (8.0)	0.206	17.52 (6.8)	18.61 (8.1)	17.84 (8.9)	0.704
**FBS** (mg/dL)	101.6 (18.6)	111.9 (38.0)	104.3 (23.9)	0.104	113.4 (36.2)	122.5 (47.5)	111.7 (29.1)	0.169	100.0 (19.7)	94.0 * (16.1)	92.6 * (16.7)	0.001	101.9 (26.3)	108.6 (40.0)	102.6 (30.2)	0.072

T0 = baseline, T1 = 12 weeks, T2 = 24 weeks. BR = Black rice germ and bran alone group, EX = Exercise alone group, BR + EX = Combined group, and CTRL = Control group. * *p* < 0.05 vs. baseline (Statistical significance using repeated measured ANOVA).

**Table 4:** Changes in general blood-based health profile parameters (lipid profile, LDL: HDL- and cholesterol: HDL-ratio) in all 4 different intervention groups during the 24-week intervention period.

Parameter	Mean (SD)															
	BR Group (*n* = 30)				EX Group (*n* =30)				BR + EX Group (*n* =28)				CTRL Group (*n* = 32)			
	T0	T1	T2	*p*-Value	T0	T1	T2	*p*-Value	T0	T1	T2	*p*-Value	T0	T1	T2	*p*-Value
**Total Cholesterol**	184.9 (42.6)	194.7 (42.1)	179.7 * (36.6)	0.001	207.7 (42.0)	209.1 (40.7)	205.2 (35.1)	0.664	210.6 (49.5)	211.8 (56.4)	200.4 (57.9)	0.088	201.9 (44.4)	212.1 (47.1)	197.8 (44.4)	0.151
**Triglycerides** (mg/dL)	115.7 (47.9)	106.3 (37.2)	95.8 * (29.7)	0.020	147.5 (101.6)	157.0 (97.2)	145.4 (93.7)	0.688	133.2 (62.0)	140.6 (72.2)	123.5 (52.0)	0.288	126.0 (54.6)	124.6 (45.3)	111.2 (57.7)	0.111
**HDL-C** (mg/dL)	52.13 (12.2)	54.53 (12.0)	52.17 (11.6)	0.109	49.63 (11.1)	49.73 (12.4)	50.50 (12.0)	0.714	55.86 (9.8)	55.79 (11.6)	53.93 (11.5)	0.295	52.06 (10.6)	56.16 (11.8)	54.6 (10.7)	0.811
**LDL-C** (mg/dL)	109.6 (35.0)	108.9 (37.0)	108.4 (32.0)	1.000	128.6 (30.8)	127.9 (35.0)	125.6 (32.4)	0.742	128.1 (45.5)	127.8 (50.4)	121.8 (53.3)	0.434	124.6 (38.4)	131.0 (41.4)	120.9 (38.3)	0.092
**LDL: HDL** Ratio	2.12 (0.58)	2.14 (0.67)	2.14 (0.64)	0.346	2.11 (0.89)	2.14 (1.09)	2.16 (0.93)	0.279	2.15 (0.82)	2.16 (0.93)	2.20 (0.95)	0.813	2.13 (0.73)	2.18 (0.80)	2.24 * (0.68)	0.007
**TC: HDL** Ratio	3.61 (0.66)	3.66 (0.79)	3.53 (0.72)	0.154	4.38 (1.38)	4.26 (1.51)	4.28 (1.27)	0.375	3.67 (1.01)	3.71 (1.16)	3.69 (1.03)	0.541	3.68 (0.82)	3.85 (0.91)	3.95 * (0.91)	0.006

T0 = baseline, T1 = 12 weeks, T2 = 24 weeks. BR = Black rice germ and bran alone group, EX = Exercise alone group, BR + EX = Combined group and CTRL = Control group. * *p* < 0.05 vs. baseline (Statistical significance using repeated measured ANOVA).

**Table 5:** Changes in aging and immunosenescence biomarkers in all 4 different intervention groups during the 24-week intervention period. (T0 = baseline, T1 = 12 weeks, T2 = 24 weeks.).

Parameter	Mean (SE)															
	BR Group (*n* = 30)				EX Group (*n* = 30)				BR + EX Group (*n* = 28)				CTRL Group (*n* = 32)			
	T0	T1	T2	*p*-Value	T0	T1	T2	*p*-Value	T0	T1	T2	*p*-Value	T0	T1	T2	*p*-Value
**IL-6** (*ρ*g/mL)	21.67 (11.03)	13.79 (7.00)	13.71 (5.50)	0.058	12.89 (4.76)	10.00 (2.40)	10.73 (2.70)	0.604	27.02 (9.66)	34.11 (7.52)	16.79 * (10.21)	0.038	17.60 (0.88)	15.25 (0.97)	17.39 (1.25)	0.641
**CRP** (mg/L)	0.97 (0.32)	0.32 (0.09)	0.23 * (0.07)	0.037	0.47 (0.08)	0.42 (0.08)	0.21 * (0.05)	0.04	0.80 (0.58)	0.56 * (0.10)	0.44 * (0.08)	<0.001	0.44 (0.08)	0.35 (0.07)	0.34 (0.05)	0.167
**IGF-1** (ng/mL)	29.17 (0.66)	41.56 * (0.39)	46.93 * (3.01)	<0.001	28.85 (1.59)	28.41 (1.24)	39.89 * (0.63)	<0.001	27.22 (1.39)	26.59 (1.29)	39.28 * (0.78)	<0.001	31.75 (0.66)	32.24 (1.40)	33.81 (4.98)	0.294
**%CD4 T cell**	35.39 (1.24)	34.58 (1.33)	35.73 (1.44)	0.291	37.69 (1.64)	35.85 (1.43)	37.11 (1.43)	0.076	31.91 (1.84)	33.48 (1.79)	34.43 * (1.75)	0.034	36.05 (1.52)	34.55 (1.24)	36.78 (1.43)	0.118
**%CD8 T cell**	18.09 (1.40)	17.85 (1.34)	19.12 (1.59)	0.103	20.34 (1.18)	20.21 (1.37)	20.59 (1.44)	0.699	19.67 (1.21)	19.38 (1.07)	19.61 (1.17)	0.800	18.96 (1.16)	18.43 (1.12)	19.33 (1.16)	0.124
**CD4:CD8 Ratio**	2.33 (0.20)	2.29 (0.21)	2.24 (0.20)	0.654	2.09 (0.19)	2.07 (0.18)	2.10 (0.18)	0.872	1.97 (0.23)	2.06 (0.27)	2.06 (0.27)	0.402	2.24 (0.22)	2.17 (0.19)	2.22 (0.21)	0.645

BR = Black rice germ and bran alone group, EX = Exercise alone group, BR + EX = Combined group and CTRL= Control group. * *p* < 0.05, ** *p* < 0.001 vs. baseline (Statistical significance using repeated measured ANOVA).

**Table 6:** Changes in physical performances and muscle strength parameters in all 4 different intervention groups during the 24-week intervention period (T0 = baseline, T1 = 12 weeks, T2 = 24 weeks).

Parameter	Median [Q1, Q3]											
	BR Group (*n* = 30)			EX Group (*n* = 30)			BR + EX Group (*n* = 28)			CTRL Group (*n* = 32)		
	T0	T1	T2	T0	T1	T2	T0	T1	T2	T0	T1	T2
**Sit to stand, Second (s)**	11.93 [8.8, 13.3]	13.08 [11.1, 18.0]	10.77 [8.2, 14.2]	14.43 [12.2, 17.2]	9.26 ** [7.3, 10.7]	8.40 ** [7.2, 10.1]	13.89 [10.7, 16.9]	8.99 ** [7.8, 10.6]	9.23 ** [7.0, 9.2]	12.12 [10.6, 13.9]	16.15 [11.6, 18.7]	12.66 [9.9, 14.3]
**Gait speed, Second (s)**	4.52 [4.2, 5.8]	5.18 [4.7, 6.2]	4.37 [3.9, 6.3]	5.43 [4.2, 7.2]	4.67 * [4.1, 6.5]	5.34 [4.4, 6.7]	5.35 [4.4, 7.5]	4.60 * [4.0, 5.3]	4.76 * [3.9, 6.6]	5.23 [4.0, 7.5]	5.38 [4.8, 6.9]	5.23 [4.2, 6.1]
**Grip strength,(kg)**	22.31 [15.9, 27.3]	18.43 [16.0, 23.7]	19.83 [17.7, 25.3]	21.13 [14.4, 25.5]	21.26 [18.0, 27.7]	22.45 [16.5, 30.2]	18.95 [13.8, 26.7]	20.75 [17.7, 26.0]	20.70 [16.3, 26.7]	18.33 [16.3, 22.9]	18.58 [16.8, 22.4]	19.46 [16.6, 25.5]

BR = Black rice germ and bran alone group, EX = Exercise alone group, BR + EX = Combined group, and CTRL = Control group. * *p* < 0.05, ** *p* < 0.001 vs. baseline (Statistical significance using Friedman test).
